# From chemolithoautotrophs to electrolithoautotrophs: CO_2_ fixation by Fe(II)-oxidizing bacteria coupled with direct uptake of electrons from solid electron sources

**DOI:** 10.3389/fmicb.2015.00994

**Published:** 2015-09-25

**Authors:** Takumi Ishii, Satoshi Kawaichi, Hirotaka Nakagawa, Kazuhito Hashimoto, Ryuhei Nakamura

**Affiliations:** ^1^Department of Applied Chemistry, School of Engineering, The University of TokyoTokyo, Japan; ^2^Biofunctional Catalyst Research Team, RIKEN Center for Sustainable Resource ScienceSaitama, Japan

**Keywords:** extracellular electron transfer, hydrothermal vents, iron oxidizing bacteria, carbon assimilation, electrolithoautotrophy

## Abstract

At deep-sea vent systems, hydrothermal emissions rich in reductive chemicals replace solar energy as fuels to support microbial carbon assimilation. Until recently, all the microbial components at vent systems have been assumed to be fostered by the primary production of chemolithoautotrophs; however, both the laboratory and on-site studies demonstrated electrical current generation at vent systems and have suggested that a portion of microbial carbon assimilation is stimulated by the direct uptake of electrons from electrically conductive minerals. Here we show that chemolithoautotrophic Fe(II)-oxidizing bacterium, *Acidithiobacillus ferrooxidans*, switches the electron source for carbon assimilation from diffusible Fe^2+^ ions to an electrode under the condition that electrical current is the only source of energy and electrons. Site-specific marking of a cytochrome aa3 complex (aa3 complex) and a cytochrome bc1 complex (bc1 complex) in viable cells demonstrated that the electrons taken directly from an electrode are used for O_2_ reduction via a down-hill pathway, which generates proton motive force that is used for pushing the electrons to NAD^+^ through a bc1 complex. Activation of carbon dioxide fixation by a direct electron uptake was also confirmed by the clear potential dependency of cell growth. These results reveal a previously unknown bioenergetic versatility of Fe(II)-oxidizing bacteria to use solid electron sources and will help with understanding carbon assimilation of microbial components living in electronically conductive chimney habitats.

## Introduction

Chemolithoautotrophs are a class of organisms that conserve their energy, electrons, and carbon from inorganic chemical sources. As opposed to phototrophs that harvest energy from the sun, they are able to synthesize their own organic molecules from the fixation of carbon dioxide under the complete absence of solar radiation. The discovery of deep-sea hydrothermal vents has revealed the physiologically and phylogenetically diverse life around the vents (Takai and Horikoshi, 1999; Reysenbach and Shock, 2002; Takai et al., 2013), and the existence of chemolithoautotroph-dependent ecosystems has sparked interest in determining the unexplored bioenergetic underpinnings of their energy yielding and carbon assimilation metabolisms (Takai et al., 2006; Baaske et al., 2007; Martin et al., 2008; Meierhenrich et al., 2010; Nakamura et al., 2010; Sander and Koschinsky, 2011; Girguis and Holden, 2012; Sievert and Vetriani, 2012; Yamamoto et al., 2013).

In such deep-vent systems, the hydrothermal fluids abundant with reductive chemicals such as H_2_, H_2_S, and Fe^2+^ are formed through high-temperature seawater-rock interaction (Takai and Horikoshi, 1999; Reysenbach and Shock, 2002; Takai et al., 2013). Considering that chemolithoautotrophs are able to conserve energy by coupling oxidation of the reductive hydrothermal fluid and reduction of the oxidative sea water containing sulfate, nitrate, and oxygen, etc., it is generally believed that nearly all microbial populations and ecosystems around deep-sea hydrothermal vents are fostered by existing diffusible reductive chemicals as energy and electron sources (Baaske et al., 2007; Sander and Koschinsky, 2011). While the validity of this conceptual framework has been well established, important details regarding the bioenergetics of microbial energy yield from geothermal sources remain open to question (Nakamura et al., 2010; Girguis and Holden, 2012; Sievert and Vetriani, 2012; Yamamoto et al., 2013; Yamaguchi et al., 2014).

Noteworthy, a recent finding of geo-electrical current generation across a wall of black-smoker chimney pointed to “electrical current flow” as a new way of energy transport from hydrothermal fluid to seawater (Nakamura et al., 2010; Yamamoto et al., 2013). Since electrical current is triggered by different redox potential of spatially segregated two redox couples, energetics that underpins energy transport to microbial niches profoundly differs from the commonly accepted notion of mass transfer, that is, energy propagation is driven by diffusion and convection of soluble reductive molecules (Baaske et al., 2007; Sander and Koschinsky, 2011). Moreover, other important findings (Nealson and Saffarini, 1994; Myers and Myers, 1997; Newman and Kolter, 2000; Gorby et al., 2006; Fredrickson et al., 2008; Marsili et al., 2008; Nakamura et al., 2009, 2013; Coursolle et al., 2010; Lovley, 2012; Mogi et al., 2013; Okamoto et al., 2013, 2014; Bose et al., 2014) of microbial extracellular electron transfer to/from metallic and/or semiconductive minerals have encouraged us to propose “electrolithoautotrophs” as the third type of microbial energy yielding metabolisms which can utilize carbon dioxide to synthesize organic matters by using electrons directly taken from solid-inorganic electron donors. According to these findings, here we propose a hypothesis that not only the diffusible reductive compounds, but also the high-energy electrons directly transported from the inner hydrothermal fluid through mineral conduit, may serve as a primary energy source for microbial ecosystems in the deep ocean (Nakamura et al., 2010; Yamamoto et al., 2013).

To examine the validity of the hypothetical metabolic pathway for electrolithoautotrophic carbon assimilation, herein we cultivated the chemolithoautotrophic Fe(II)-oxidizing bacterium, *Acidithiobacillus ferrooxidans*, in Fe^2+^-ions free electrochemical reactors. Using site-specific chemical marking for intracellular electron-transfer chains involved in carbon assimilation, we demonstrate the previously unaccounted ability of an Fe(II)-oxidizing bacterium to switch the metabolic mode from chemosynthesis to hypothetical electrolithoautotrophic carbon assimilation under the conditions that electrical current is the only source of energy and electrons for their carbon assimilation.

## Materials and Methods

### Cell Preparation

*Acidithiobacillus ferrooxidans* (ATCC23270) was cultured in DSMZ medium 882 (132 mg L^-1^ (NH_4_)_2_SO_4_, 53 mg L^-1^ MgCl_2_6H_2_O, 27 mg L^-1^ KH_2_PO_4_, 147 mg L^-1^ CaCl_2_2H_2_O, and trace elements) supplemented with ferrous iron (66 mM) as an electron source and incubated aerobically at 30°C with shaking at 150 rpm in Erlenmeyer flask (volume of medium: 150 mL). The pH of solutions was adjusted to 1.8 using 5 M H_2_SO_4_. Subsequently, the culture was centrifuged at 15000 rpm for 10 min, and the pelleted cells were washed vigorously with a fresh medium at pH 1.8. This process was repeated more than three times to remove soluble Fe^2+^ ions and insoluble iron oxides from the cell culture prior to being used for electrochemical experiments.

### Electrochemical Measurements

A single-chamber three-electrode system equipped with the working electrode on the bottom surface of the reactor was used for the electrochemical analysis of intact cells. A conducting glass substrate [fluorine-doped tin oxide (FTO)-coated glass electrode, resistance: 20 Ω/square, size: 30 mm × 30 mm; SPD Laboratory, Inc.] was used as the working electrode. The reference and counter electrodes were Ag/AgCl (KCl sat.) and a platinum wire, respectively. An air-exposed DSMZ medium 882 was used as an electrolyte. The pH of solutions was adjusted to 1.8 using 5 M H_2_SO_4_. The head space of the reactor was purged with air which is the source of N_2_, O_2_, and CO_2_.

### Chemical Marking Experiments

Coordination of CO to heme proteins in living cells was carried out by bubbling the cell suspension of *A. ferrooxidans* with CO gas for 10 min in the electrochemical reactor (Shibanuma et al., 2011). For the photocurrent measurements, a 1000-w Xe lamp (Ushio) equipped with a monochromator with a band width of 10 nm was used as an excitation source to irradiate light from the bottom of the electrochemical cell. For the inhibitor experiment of a bc1 complex, 1 v/v % Antimycin A solubilized in methanol was added in the electrochemical reactor. The final concentration of Antimycin A was 100 μM.

## Results and Discussion

### Branched Electron-Transfer Chain of *A. ferrooxidans*

**SCHEME 1:**
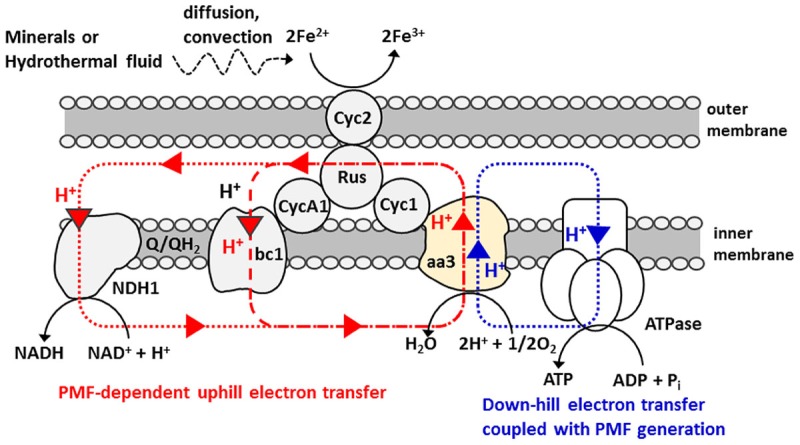
**Bifurcated electron and proton transfer model of Fe(II) oxidation in *Acidithiobacillus ferrooxidans* (Sugio et al., 1981; Ingledew, 1982; Elbehti et al., 1999, 2000; Brasseur et al., 2004; Valdés et al., 2008; Quatrini et al., 2009; Bird et al., 2011).** A small periplasmic blue copper protein (rusticyanin, Rus) has been proposed as a branch point to switch an electron flow between NAD^+^ and O_2_. Proton circuit for a down-hill and an up-hill electron-transfer reaction is indicated by blue and red dotted line, respectively. Electron and energy delivery to the cells for carbon fixation is based on the diffusion and/or convection of soluble Fe^2+^ ions.

The electron transfer pathways spanning from the outer to inner-membrane of *A. ferrooxidans* has been extensively studied and the bifurcated chain composed of “down-hill (exergonic)” and “up-hill (endergonic)” pathways has been identified (**Scheme 1**) (Sugio et al., 1981; Ingledew, 1982; Elbehti et al., 1999, 2000; Yarzábal et al., 2002; Brasseur et al., 2004; Valdés et al., 2008; Quatrini et al., 2009; Bird et al., 2011; Shibanuma et al., 2011). Of particular note is that like *Shewanella* and *Geobacter* species known to have an ability for the direct extracellular electron transfer to and from an electrode, *A. ferrooxidans* also has *c-*type cytochromes (Cyc 2) on their outer-membrane compartments (Yarzábal et al., 2002). Electrons gained by Fe^2+^ oxidation at Cyc 2 are used for O_2_ reduction via a down-hill pathway, which in turn generates proton motive force (PMF) that is used for pushing the electron to a bc1 complex via an up-hill pathway and/or triggering ATP synthesis (Sugio et al., 1981; Ingledew, 1982; Elbehti et al., 1999, 2000; Yarzábal et al., 2002; Brasseur et al., 2004; Valdés et al., 2008; Quatrini et al., 2009; Bird et al., 2011; Shibanuma et al., 2011). In the following experiments, *A. ferrooxidans* was inoculated in an electrochemical reactor without Fe^2+^ ions and examined if the PMF-dependent up-hill pathway is activated by the direct electron uptake from an electrode, instead of the oxidation of diffusible Fe^2+^ ions.

### Direct Uptake of Electrons from an Electrode into Cells

**FIGURE 1:**
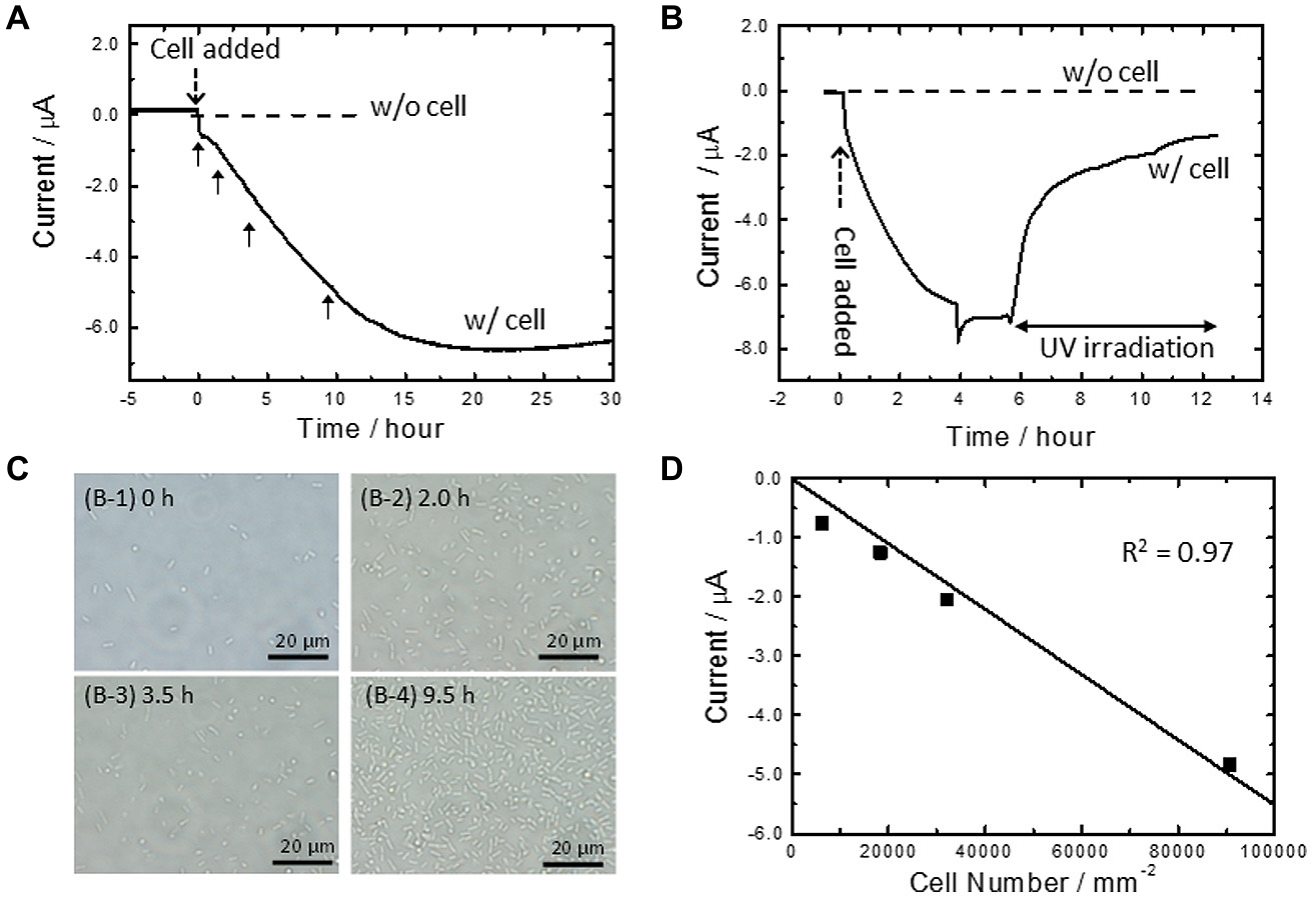
**(A)** Current vs. time measurements for microbial current generation by *Acidithiobacillus ferrooxidans* cells on an fluorine-doped tin oxide (FTO) electrode in the absence of Fe^2+^ ions (solid line) at +0.4 V (vs. SHE). Current vs. time measurements without cells at +0.4 V was also depicted as a reference (broken line) **(B)** Effects of the deep-UV (254 nm) irradiation to the microbial current generation by the cells in the absence of Fe^2+^ ions at +0.4 V (solid line). Current vs. time measurements without cells at +0.4 V was also depicted as a reference (broken line). **(C)** *In-situ* optical microscope observation of an FTO electrode surface at the indicated time (panel A) after cell inoculation. **(D)** Plot of microbial current against cell number attached on an electrode surface obtained from *in-situ* optical microscope observation (panels A and B). The squares of the correlation coefficients were estimated by the addition of the point of origin to the obtained data. The geometric area of the FTO electrode was 3.14 cm^2^. Initial OD_500_ was 0.02.

**Figure 1A** shows current vs. time curves for *A. ferrooxidans* cultivated in the absence of Fe^2+^ ions. In the present system, a conducting glass electrode (FTO) poised at +0.4 V (vs. SHE) acts as a sole source of electrons, and dissolved O_2_ and CO_2_ are an electron acceptor and a carbon source, respectively. In the absence of bacteria, we detected no electrical current generation (broken line, **Figure 1A**). On the other hand, in the reactors containing cells, the cathodic current gradually increased to approximately 7 μA after 20 h of cultivation (solid line, **Figure 1A**). The marked difference in current density depending on the presence of cells indicates that the cathodic current was derived from the metabolic activity of cells. Furthermore, *in-situ* sterilization of cells with the deep-UV (254 nm) irradiation immediately suppressed the cathodic current generation (**Figure 1B**). Almost no electrical response was observed after 6 h of sterilization, confirming the strong coupling of metabolic activity to electrical current generation.

It is noted that the cathodic current generation by *A. ferrooxidans* in **Figure 1A** is mostly derived from the cells attached on an electrode, rather than planktonic cells. This was confirmed by *in-situ* counting of cell number on the FTO electrode with simultaneous monitoring of electrical current generation (**Figures 1C,D**). Since an FTO electrode is optically transparent and placed on the bottom surface of the electrochemical reactor, optical microscope images of the electrode surface can be acquired during electrical current generation by *A. ferrooxidans*. **Figure 1C** shows the *in-situ* optical microscope observation of the FTO electrode surface at the indicated time in **Figure 1A** after the cell was added in the electrochemical reactor. We plotted current against cell number to quantify the contribution of the electrode-attached cells for the cathodic current generation. As shown in **Figure 1D**, the microbial current exhibited a negative correlation with the cell number, as a fitted line passed through the point of origin with a high correlation coefficient (*r*^2^ = 0.97). This means that the current production was indeed dominated by the extracellular electron transfer of the cells directly attaching on the electrode surface. In other words, it appears that the gradual increase in the cathodic current in **Figure 1A** correlates with the establishing process of the electrical conduits to the electrode poised at +0.4 V with the cells settled down to the bottom part of the reactor.

**FIGURE 2:**
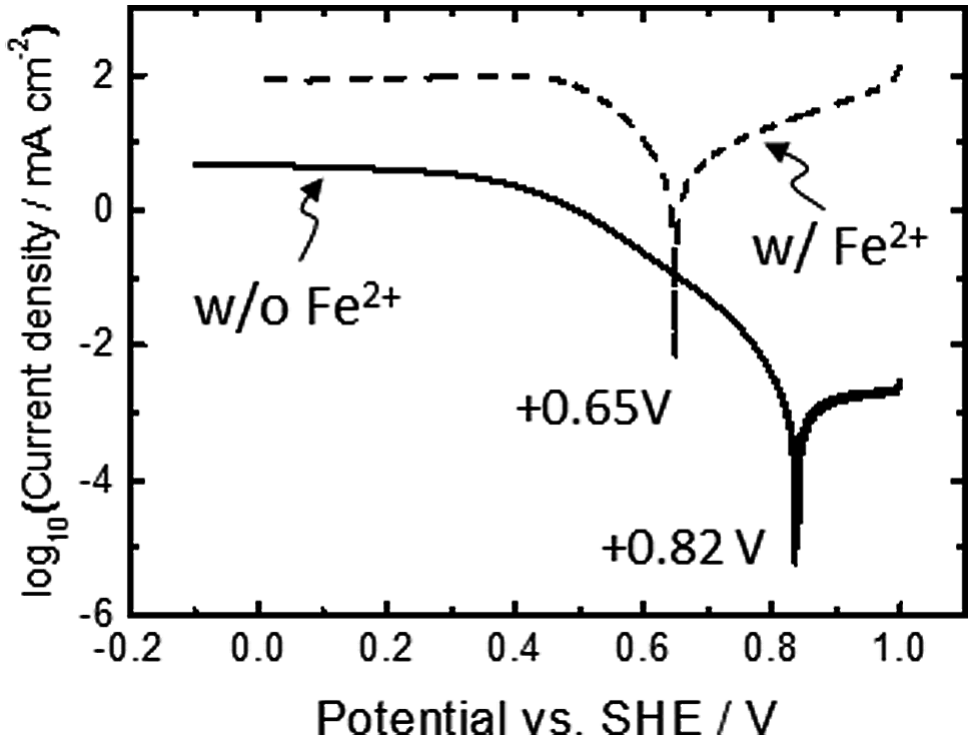
**Linear sweep (LS) voltammograms for *A. ferrooxidans* cultivated in the presence (broken line) and absence (solid line) of Fe^2+^ ions (66 mM).** A scan rate was 0.1 mV s^-1^. Initial OD_500_ was 0.02.

To evaluate the redox potential of electrical conduits established at the cell-electrode interface, electrodes covered with viable cells were examined by linear sweep (LS) voltammetry (solid line, **Figure 2**). As a reference, we also conducted LS voltammetry for cell cultures containing soluble Fe^2+^ ions, a system known as electrochemical cultivation of *A. ferrooxidans* (broken line, **Figure 2**) (Yunker and Radovich, 1986; Matsumoto et al., 1999, 2002; Ishii et al., 2012). In the presence of Fe^2+^ ions, the onset potential for cathodic current generation was estimated to be +0.65 V, as indicated in the peak in the logarithmic plot for current. This value is close to the redox potential of Fe^3+^/Fe^2+^ couple and consistent well with the previously reported model for electrochemical cultivation of *A. ferrooxidans* (Yunker and Radovich, 1986; Matsumoto et al., 1999, 2002; Ishii et al., 2012). Namely, diffusible Fe^2+^ and Fe^3+^ ions serve as an electron shuttle which bridges electron transfer between planktonic cells and electrodes. Meanwhile, the LS voltammogram for the electrode-attaching cells provided one peak at +0.82 V, and no peak assignable to diffusible Fe^3+^/Fe^2+^ redox couple was observed. This is the clear indication that the electrode-attaching cells established the different conduit of electrons to the FTO electrode rather than the Fe^3+^/Fe^2+^ redox couple, which serves as an important information for understanding the bioenergetics of PMF-dependent electrolithoautotrophic carbon assimilation (discuss later). Since the cathodic current generation was dominated by the cells directly attaching on an electrode surface (**Figure 1D**), the LS voltammetry results suggest the existence of outer-membrane-bound redox protein at a midpoint potential of +0.82 V, which bridges an electron donor of an FTO electrode to inner electron-trasnsfer chains responsible for carbon fixation.

### *In-Vivo* Monitoring Down-Hill Electron-Transfer Pathway

To identify the inner electron-trasnsfer chains responsible for the cathodic current generation and to examine if the PMF-dependent up-hill pathway is activated by direct uptake of electrons from an electrode, we applied the artificial photochemical reaction to *A. ferrooxidans*. As previously reported (Shibanuma et al., 2011), the treatment of viable cells with CO allows for monitoring the electron-transfer reaction mediated by heme proteins under *in-vivo* conditions, since the redox activities of hemes are blocked upon CO binding and subsequently reactivated by photodissociation of CO. This technique enables us to identify the specific heme proteins involved in current generation by examining the wavelength dependency of photocurrent response. In this experiment, *A. ferrooxidans* cells inoculated in an Fe^2+^-free electrochemical reactor were treated with CO and irradiated by the monochromic light with a band width of 10 nm in a course of microbial current generation.

**FIGURE 3:**
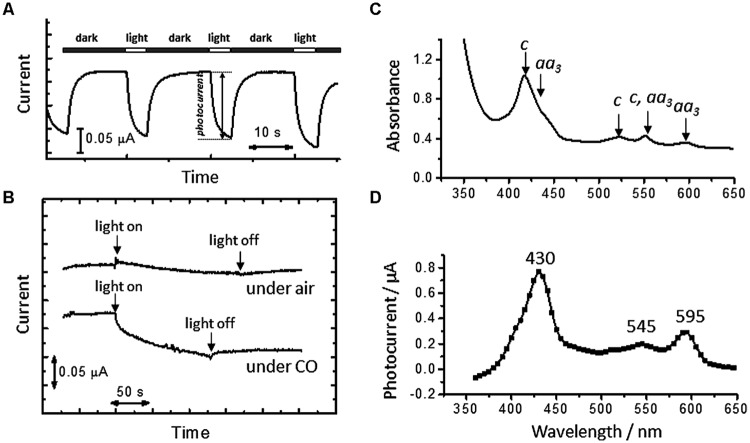
**(A)** Time courses of microbial current generation in the absence of Fe^2+^ ions under CO atmospheres. White and black bars indicate the period for light irradiation and dark conditions, respectively. An electrode potential was +0.4 V (vs. SHE). **(B)** Time courses of microbial current at an electrode potential of +0.4 V under air and CO atmospheres. **(C)** Diffuse transmission UV-vis spectrum of whole cells of *A. ferrooxidans* suspended in a DSMZ medium containing Na_2_S_2_O_4_ as a reductant under CO atmospheres. **(D)** An action spectrum of the microbial current recovered by visible-light irradiation under a CO atmosphere. Initial OD_500_ was 0.02.

**Figure 3A** shows time courses of microbial current at an electrode potential of +0.4 V under CO atmospheres. Upon the treatment of the cells with CO, the microbial current decreased, suggesting that the formation of CO-ligated heme in living cells inhibited the extracellular electron-transfer reactions of *A. ferrooxidans*. The drop in the microbial current caused by CO was recovered upon visible-light irradiation; in contrast, visible-light irradiation induced little change in the current generation under N_2_ atmospheres (**Figure 3B**).

A whole cell of *A. ferrooxidans* has multiple kinds of heme proteins, as indicated by UV-visible spectra of cell suspensions under CO atmospheres in the presence of Na_2_S_2_O_4_ (50 mM) as a reductant (**Figure 3C**). It is seen from the spectral region of Q bands that the cell has *a*-type (∼550 and ∼600 nm) and *c*-type (∼525 and ∼550 nm) cytochromes. To clarify the origin of the light-induced current recovery, excitation wavelength dependency was investigated. The action spectrum of CO-treated cells resolved the three bands peaked at 430, 545, and 595 nm, which are well-correlated with the Soret (429 nm) and Q (546 and 590 nm) bands of a CO-ligated aa3 complex, respectively (**Figure 3D**) (Horie et al., 1983). This indicates that the current recovery due to visible-light irradiation is predominantly derived from the photodissociation of the CO ligand of an aa3 complex. Namely, the photodissociation generates redox-active hemes and revives the cellular respiratory electron transport reactions. The suppression of microbial current observed immediately after stopping visible-light irradiation (**Figure 3A**) is due to the recombination of CO with an aa3 complex.

### *In-Vivo* Monitoring of up-Hill Electron Transfer Pathway

**FIGURE 4:**
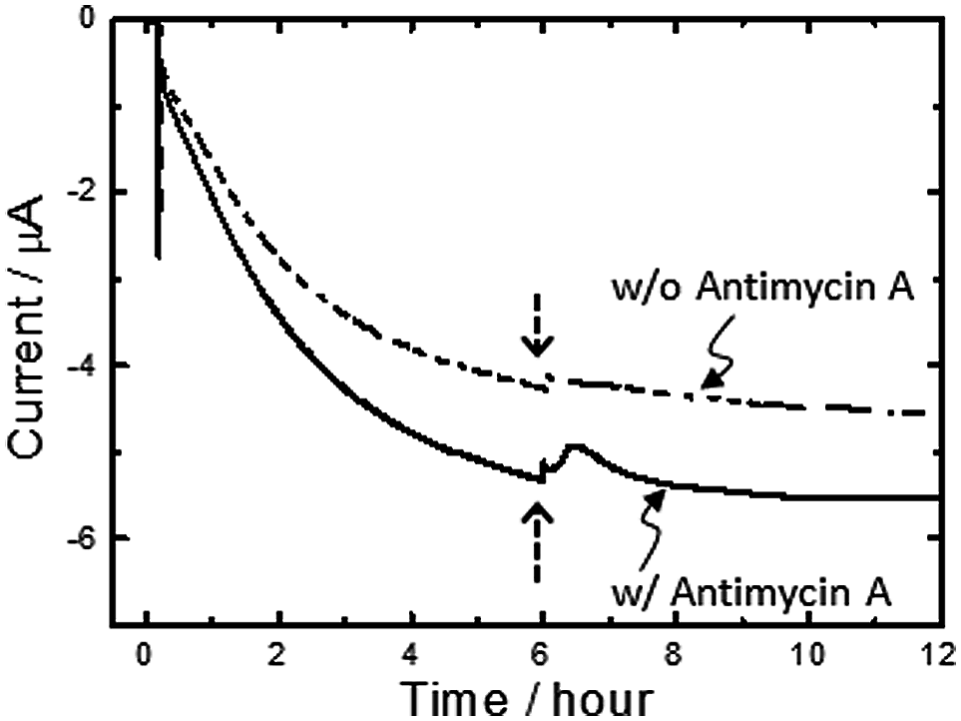
**Effects adding a bc1 complex inhibitor, Antimycin A, on microbial current generation for *A. ferrooxidans* cultivated in the absence of Fe^2+^ ions at an electrode potential of +0.4 V (vs. SHE).** Antimycin A solubilized in methanol (final concentration of Antimycin A is 100 μM) was added into electrochemical reactors at the time points indicated with an arrow (solid line). Methanol lacking Antimycin A was also added into electrochemical reactors as a control experiment (broken line). Initial OD_500_ was 0.02.

It is worth noting here that the aa3 complex of *A. ferrooxidans* is expressed under Fe^2+^-grown conditions and positioned at a terminal of the down-hill pathway (**Scheme 1**) (Sugio et al., 1981; Ingledew, 1982; Elbehti et al., 1999, 2000; Yarzábal et al., 2002; Brasseur et al., 2004; Valdés et al., 2008; Quatrini et al., 2009; Bird et al., 2011). Given that an aa3 complex is responsible for the generation of PMF for up-hill pumping of electrons and/or initiating ATP synthesis, it can be deduced that a portion of electrons directly taken from an electrode are pushed up-hill to NAD^+^ through a bc1 complex. To assure the occurrence of PMF-dependent reverse electron transfer, we added a bc1 complex inhibitor, Antimycin A (Elbehti et al., 2000), to the electrode-attaching cells in the course of microbial current generation. As expected, upon addition of 1v/v % Antimycin A solubilized in methanol (final concentration of Antimycin A is 100 μM), the transient, but clear suppression of the microbial current generation by approx. 6% was observed (solid line, **Figure 4**). In contrast, the addition of 1 v/v % methanol lacking Antimycin A caused subtle change in the microbial current (broken line, **Figure 4**), confirming that the current suppression was due to the inhibition of the bc1 complex.

**FIGURE 5:**
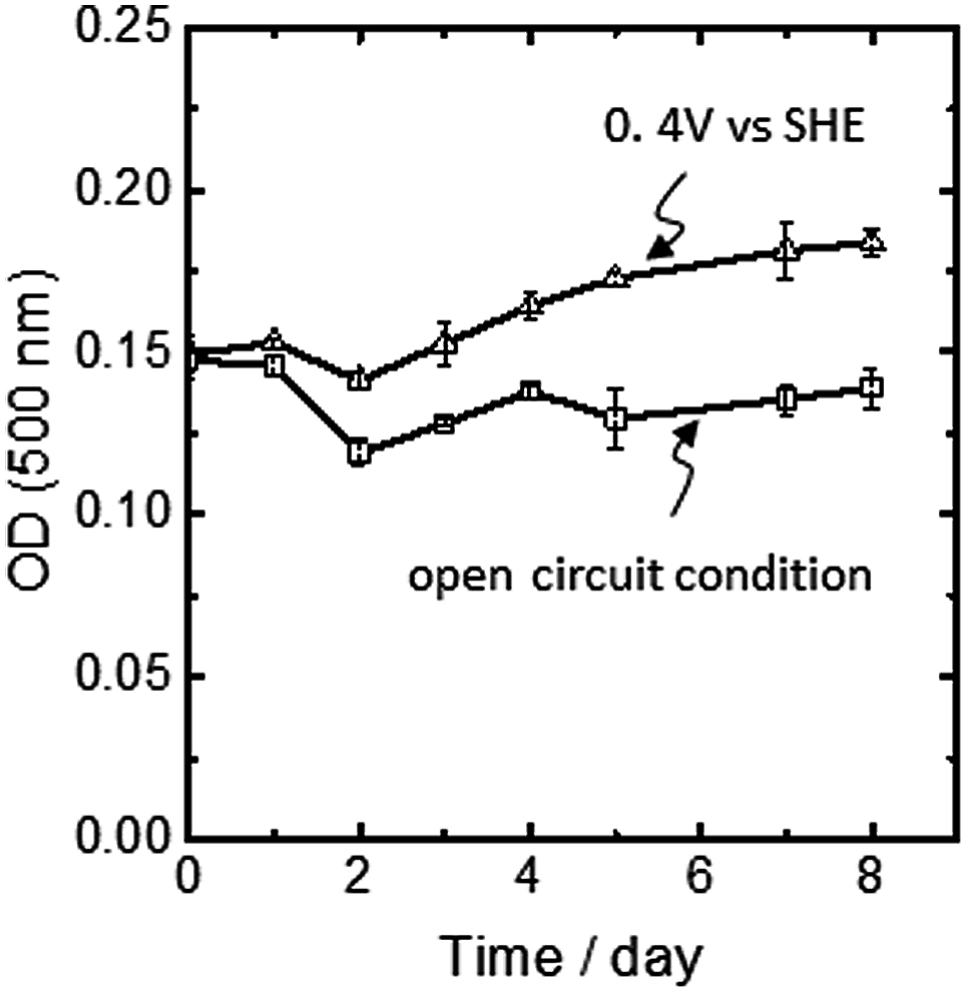
**Changes in the optical cell density at 500 nm of *A. ferrooxidans* inoculated in an Fe^2+^-ion free electrochemical rector under the potential static condition at +0.4 V vs. SHE (open triangle) and the open circuit condition (open square).** Error bars indicate the standard error of the means calculated with data obtained from three individual experiments for the potential static condition and two individual experiments for the open circuit condition, respectively.

In *A. ferrooxidans*, the PMF-dependent up-hill electron transfer is a physiologically important phenomenon, since carbon dioxide fixation via the Calvin cycle is coupled to this process. **Figure 5** shows the time course of optical cell density at 500 nm (OD_500_) obtained for *A. ferrooxidans* cells inoculated in an Fe^2+^-ion-free electrochemical reactor for 8 days. Under the condition that the electrode potential was poised at +0.4 V, OD_500_ increased with incubation time. In contrast, when the cell was incubated under the same condition with the exception that no external potential was applied to the FTO electrode (open circuit condition), no growth of the cells was observed. Here, we should emphasize that under the open circuit condition, the electron flow from the FTO electrode to the cells was fully ceased and thus the electrode no longer functioned as an electron source for microbial growth. Therefore, the clear potential dependency of the cell growth indicates that the cathodic current was being used not only for PMF generation, but also for carbon dioxide fixation and cellular maintenance via an endergonic electron-transfer reaction.

**SCHEME 2:**
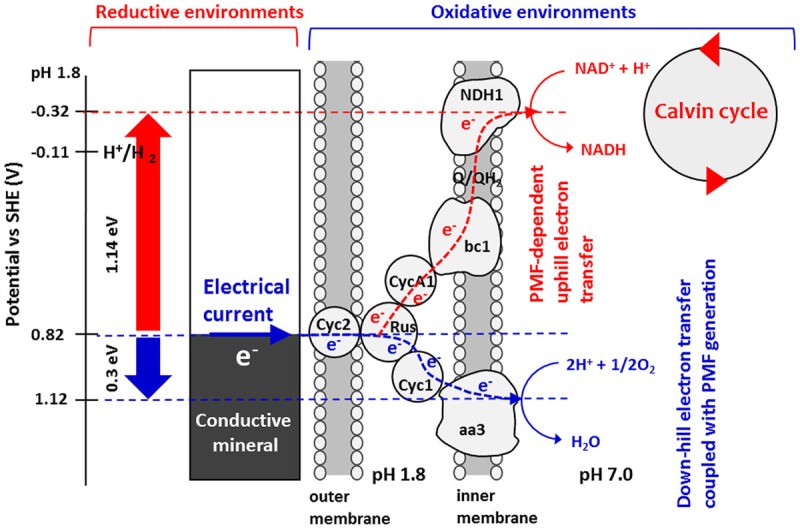
**Energy diagram for PMF-dependent electrolithoautotrophic carbon fixation in *A. ferrooxidans*.** *A. ferrooxidans* cell extracts electrons directly from solid electron sources such as conductive minerals and electrodes at a potential of +0.82 V vs. SHE. Electrons are used for O_2_ reduction via a down-hill pathway, which in turn generates PMF that is used to elevate the energy of electrons to reduce NAD^+^ to NADH, therefore triggering Calvin cycle. From the energy difference between the input electron and the midpoint potential of NAD+/NADH redox at cytoplasmic pH, it is estimated that *A. ferrooxidans* elevates the energy of electrons using PMF by 1.14 eV.

Taken together, the results obtained by *in-vivo* electrochemistry with site-specific chemical marking suggest the bioenergetic pathway illustrated in **Scheme 2** as a model for PMF-dependent electrolithoautotrophic carbon assimilation of *A. ferrooxidans*. As estimated from LS voltammetry (**Figure 2**), *A. ferrooxidans* cells establish the direct electrical conduit to a solid electron donor at the potential of +0.82 V, which is 0.17 V more positive than the diffusible redox couple of Fe^3+^/Fe^2+^ for chemolithoautotrophic carbon assimilation. Although Cyc 2 is located at the outer-cell surface of *A. ferooxidance*, we cannot exclude the possibility that self-secreted redox molecules are involved in the direct extracellular electron transfer, as recent studies demonstrated that self-secreted flavin acts as a bound-cofactor of outer-membrane *c*-type cytochromes of *Shewanella oneidensis* (Okamoto et al., 2013) and *Geobacter sulfurreducens* (Okamoto et al., 2014) for initiating the direct electron transfer from cells to electrodes. The involvement of H_2_ as an electron carrier for the direct extracellular electron transfer and cell growth of *A. ferooxidance* can be excluded, since the onset potential of direct extracellular electron transfer is approximately 900 mV more positive than the redox potential of hydrogen evolution [*E*(H^+^/H_2_) = - 0.11 V vs. SHE at pH1.8]. Even in the presence of extracellular enzymes suggested by *Methanococcus maripaludis* (Deutzmann et al., 2015), H_2_ production by proton reduction is thermodynamically unfeasible in our experimental conditions.

For initiating the carbon fixation by Rubisco enzymes, *A. ferrooxydans* cells require sufficient reductive energy to convert NAD^+^ to NADH in the cytoplasm through a bc1 complex. As the reversible potential of the NAD^+^/NADH redox couple is -0.32 V at cytoplasmic pH, we can estimate that *A. ferrooxydans* cells are capable of elevating the energy of electrons by 1.14 V using PMF through exergonic electron flow to O_2_ (**Scheme 2**). Although how the electron flow switches between NAD^+^ and O_2_ is not known, a small periplasmic blue copper protein (rusticyanin, Rus) has been proposed as a branch point (Valdés et al., 2008; Quatrini et al., 2009). From the inhibition rate of cathodic current generation by Antimycin A (**Figure 4**), we can estimate that electrons gained from an FTO electrode split to an up-hill and a down-hill pathway with a ratio of approximately 1 to 15 under the condition of the present experiments. The presence of two functional bc1 complexes are known in *A. ferrooxidans*. One (PetA1B1C1) functions only in up-hill direction in iron-grown cells (Elbehti et al., 2000), whereas the other (PetA2B2C2) has been shown to function only in down-hill mode in sulfur-grown cells (Brasseur et al., 2004). Considering that the PMF-dependent up-hill pathway is activated for the cells attached on the electrode surface, it is assumable that the former one is responsible in the observed electrolithoautotrophic carbon assimilation.

## Summary

In the present study, we demonstrated for the first time that the chemolithoautotrophic Fe(II)-oxidizing bacterium, *A. ferrooxidans*, is capable of using electrical current as the energy source for carbon assimilation. The finding of the metabolism switched from chemolithoautotrophy to electrolithoautotrophy demonstrates a previously unknown bioenergetic versatility of energy yielding metabolisms in Fe(II)-oxidizing bacteria, which in turn supports our hypothesis (Nakamura et al., 2010; Yamamoto et al., 2013) highlighting the possibility of electrons to be a primary energy source for deep-sea hydrothermal ecosystems. Although recent studies have postulated that several microorganisms are capable of conducting a direct uptake of electrons from solid electron donors at highly negative redox potential ranging from -400 to -800 mV (vs. SHE; reviewed in Rabaey and Rozendal, 2010; Lovley and Nevin, 2013; Deng et al., 2015; Tremblay and Zhang, 2015), these potentials are too negative to be generated by natural environments, even at the highly reducing environments such as deep-sea alkaline hydrothermal vents (Martin et al., 2008). Accordingly, further investigation on PMF-dependent electrolithoautotrophy at geochemically relevant potential regions presented in this work will help understanding carbon assimilation of microbial components living around electronically conductive chimney walls. On-site electrochemical experiments at deep-sea hydrothermal vents are currently underway using a remotely operated vehicle equipped with the potentiostat and potential programmer system (Yamamoto et al., 2013), which will bring our understanding of electricity-dependent microbial habitats to a new realm.

## Conflict of Interest Statement

The authors declare that the research was conducted in the absence of any commercial or financial relationships that could be construed as a potential conflict of interest.
